# Next-Hop Relay Selection for Ad Hoc Network-Assisted Train-to-Train Communications in the CBTC System

**DOI:** 10.3390/s23135883

**Published:** 2023-06-25

**Authors:** Sixing Ma, Meng Li, Ruizhe Yang, Yang Sun, Zhuwei Wang, Pengbo Si

**Affiliations:** Faculty of Information Technology, Beijing University of Technology, Beijing 100124, China; masixing958@emails.bjut.edu.cn (S.M.); yangruizhe@bjut.edu.cn (R.Y.); sunyang@bjut.edu.cn (Y.S.); wangzhuwei@bjut.edu.cn (Z.W.); sipengbo@bjut.edu.cn (P.S.)

**Keywords:** communication-based train control (CBTC), train-to-train(T2T) communication, relay selection, multiagent dueling DQN

## Abstract

In the communication-based train control (CBTC) system, traditional modes such as LTE or WLAN in train-to-train (T2T) communication face the problem of a complex and costly deployment of base stations and ground core networks. Therefore, the multi-hop ad hoc network, which has the characteristics of being relatively flexible and cheap, is considered for CBTC. However, because of the high mobility of the train, it is likely to move out of the communication range of wayside nodes. Moreover, some wayside nodes are heavily congested, resulting in long packet queuing delays that cannot meet the transmission requirements. To solve these problems, in this paper, we investigate the next-hop relay selection problem in multi-hop ad hoc networks to minimize transmission time, enhance the network throughput, and ensure the channel quality. In addition, we propose a multiagent dueling deep Q learning (DQN) algorithm to optimize the delay and throughput of the entire link by selecting the next-hop relay node. The simulation results show that, compared with the existing routing algorithms, it has obvious improvement in the aspects of delay, throughput, and packet loss rate.

## 1. Introduction

Recently, with the rapid development of urbanization, urban rail transit has become one of the main transportations. With the development of technology, the communication-based train control (CBTC) system plays an important role in urban rail transit to guarantee the safe operation of rail trains [1]. To ensure their safety and reliability, CBTC systems have strict requirements on transmission delay and channel quality [2]. Long communication delay and link interruptions may lead to emergency brakes or collisions [3]. Therefore, it is crucial to design a CBTC communication system with low latency and high channel quality.

In traditional CBTC systems, long-term evolution for metro (LTE-M) and wireless local area networks (WLANs) are more widely used in train-to-wayside communication [4]. The train information is first transmitted to the ground-zone controller (G-ZC), which is used to generate control commands for all trains in its management area [5]. After obtaining the commands, the wayside node sends the commands back to trains. However, due to the huge computational burden of the G-ZC and non-direct transmission link [6], the transmission delay of important control commands is excessively large. Therefore, the T2T direct transmission approach was proposed [4], while the G-ZC was also changed to an onboard-zone controller (On-ZC). Unlike the G-ZC, the On-ZC only needs to generate its own commands, which greatly reduces computation latency.

Although the direct T2T transmission greatly reduces latency [7], if we continue to use WLAN or LTE, interruptions and delays caused by hard handoff at the base station boundary are still unavoidable. In addition, the deployment of the terrestrial core network and base station are complex, which makes network construction and maintenance cost high. Therefore, new technologies such as reconfigurable intelligent surfaces [8,9] and wireless ad hoc networks have been proposed to improve T2T communication.

The wireless ad hoc network is a novel approach to improve the performance of CBTC systems. In the wireless ad hoc network, the packages are sent to the wayside node from the on-board node. Similar to the vehicular ad hoc network (VANET), the role of wayside nodes is to assist packet transmission [10]. Therefore, the packages are transmitted through the transmission network formed by the wayside nodes hop-by-hop and finally to the running train, so that the transmission link is more stable. Furthermore, the deployment of wireless ad hoc network nodes is less costly, and it does not have a fixed topology or require any fixed infrastructure to communicate, allowing it to be deployed more flexibly and configured quickly.

The relay selection strategy plays an important role in wireless ad hoc networks to reduce transmission delay, improve throughput, and decrease packet loss. However, since wireless ad hoc networks are rarely used in CBTC systems, no suitable routing strategy has been proposed. Therefore, the existing strategies from VANETs and mobile ad hoc networks (MANETs) should be considered and improved to adapt to the CBTC system.

In VANET and MANET relay selection strategies, the routing algorithms are generally divided into two types: proactive routing and reactive routing [11]. Traditional proactive routing approaches, such as optimized link-state routing (OLSR) [12] and destination sequenced distance vector (DSDV) [13], require significant overhead for node exploration and maintenance of routing tables, which is not feasible for CBTC systems with strict requirements for low latency. At the same time, although the traditional reactive routing greedy perimeter stateless routing (GPSR) can directly select the next-hop relay, it cannot fully consider various factors such as node congestion and channel quality. In the CBTC system, the transmission channel can be affected by the high mobility of the train, causing shadow fading and multipath fading, which leads to a sudden change in transmission link. Therefore, the relay selection strategy must be able to make decisions according to the varying channel state.

On the basis of past training experience, the learning-based routing algorithm can make real-time decisions well by observing current channel state. Meanwhile, in deep reinforcement learning (DRL), the optimization problem of multiple factors can be transformed to maximize cumulative rewards [14]. We can combine diverse factors to design rewards, in order to achieve optimization of these indicators. Therefore, learning-based routing algorithms are more suitable for CBTC networks. Existing DRL-based algorithms are often used in relay selection for VANET and MANET [15,16]; these algorithms consider more focus on single-hop delay, outage probability, and power consumption as the criteria for next-hop selection. However, they ignore the whole-link delay and throughput.

In this paper, our objective is to design a low-latency and high-throughput routing method in an ad hoc network for a CBTC system. However, packet transmission still faces challenges in multi-hop relay selection. For example, due to the high-speed train, the transmission distance between the train and wayside node is limited. In order to decrease the outage probability, we set strict distance limitations when transmitting with trains. In addition, we comprehensively consider the transmission delay, queuing delay, and channel quality, aiming to optimize the overall performance of the link by selecting the next-hop node. Moreover, the process of selecting the next-hop node can be formulated as a Markov decision process (MDP) [17,18]. We propose a multi-agent DRL method to solve the problem. The main contributions of this paper are summarized as follows:

1. We formulate the next-hop relay selection problem in a CBTC system. The goal is to select relay nodes with low transmission delay and high throughput in both the train and the wayside node communication range. Meanwhile, in order to balance the single-hop transmission delay and the whole-link hop count, we propose the concept of “hop tradeoff” to minimize the entire link latency.
2. To handle the time-varying channel state and node congestion, we propose a DRL algorithm to optimize the long-term system reward. Using a multiagent approach [14], all nodes are trained centrally with dueling DQN [19], and then each node makes the next-hop decision individually, in order to avoid nodes with a long queuing delay and poor channel quality.
3. Lastly, we conduct simulations with a different number of nodes between two trains and different buffer sizes. Meanwhile, the proposed algorithm is compared with several existing algorithms in terms of whole-link delay, packet loss rate, and throughput. The simulation results indicate that the proposed scheme works well against congested networks. In particular, it also significantly superior to other routing algorithms in the aspects of whole-link delay, throughput, and packet loss rate.

The remainder of this paper is organized as follows: in Section 2, some related work about routing selection in ad hoc networks is introduced; in Section 3, we present a multi-hop relay selection model for ad hoc networks in CBTC systems; the joint optimization problem of channel throughput and total-link delay is formulated in Section 4; then, we introduce the multiagent deep reinforcement method to solve the formulated problem in Section 5; some simulation results and analyses are presented in Section 6; in Section 7, we conclude the paper and propose some future work.

## 2. Related Work

### 2.1. Traditional Communication Method in CBTC System

In the traditional CBTC system, WLAN is widely used in the communication between trains and the wayside base station. Zhu et al. [20] proposed a WLAN-based redundant connect scheme for train–ground communication. The train connects the backup link and active link simultaneously to deal with the interruption at the coverage boundaries of the two access nodes. However, many WLAN standards based on IEEE802.11 are not suitable for high-speed mobile environments [5]. Meanwhile, WLAN is in the open frequency band, which can easily be interfered with by other devices [21]. LTE has strong anti-interference ability and is more stable in switching between access nodes; thus, LTE-based approaches have been proposed in the CBTC system. In [6], a sensing-based semi-persistent scheduling method for LTE-based T2T communication was proposed, which greatly improved the transmission delay of system safety information. However, both LTE and WLAN have the problem of packet loss and delay due to the switch between the access nodes, in addition to the high cost of the base stations and ground core network. Since wireless ad hoc networks do not require a fixed infrastructure, their deployment is more flexible and cheaper. Therefore, ad hoc networks are also a better choice for T2T communication.

### 2.2. Traditional Ad Hoc Network Route Selection

In ad hoc network application, the packet routing is critical for optimizing transmission delay, throughput, and packet loss. There are two types of routing methods commonly used in ad hoc networks: proactive routing and reactive routing. In proactive routing, the OLSR protocol [12] is to store the information of each relay node into the routing table by sending HELLO packets in advance, and then select the shortest path from routing table. The DSDV protocol [13] uses the Bellman–Ford algorithm to select relay nodes in the routing table. Although these approaches allow the optimal route to be selected, they require a large amount of information to be exchanged among all nodes. Especially when the nodes are dynamic, this leads to a rapid increase in the amount of information exchanged. Nevertheless, in the CBTC system, the trains move rapidly, and transmission latency is strict; thus, it is more suitable to use reactive routing.

In reactive routing, nodes cannot know global information of the whole network and can only make decisions for selecting the next-hop node. In [22], the GPSR protocol was derived, and the node which is closest to the destination within the communication range was selected as next-hop node. This minimizes the number of hops in the entire link, thus reducing latency. In order to solve the high outage probability caused by the high-speed movement of vehicles, the authors of [23] proposed a hybrid relay node selection strategy. The relay with the best channel quality and the relay closest to the destination were simultaneously selected as the next-hop node. Although this method improved the transmission successful rate, the overhead of transmitting both packets simultaneously was huge.

### 2.3. The Relay Selection of Ad Hoc Network Enabled by DRL

The deep learning-based relay selection method can make a better choice for next-hop relay where transmission rate and node congestion change in real time, and it can also synthesize multiple performance factors to make comprehensive decisions. Therefore, the routing method based on learning-based algorithms performs better in mobile communication network.

Learning-based routing methods are widely used in MANETs and VANETs [24]. As the mobility of nodes may lead to constant variations in the network topology [25], the learning-based approach can make decisions according to real-time changes and is, thus, more suitable for both scenarios. In [26], Wang et al. proposed a multiagent method, wherein all agents share the same training experience based on DQN, but make the next-hop decision individually. The aim of this method is to select the optimal route in MANET, which minimizes transmission delay and queuing delay. In order to minimize delay and make a reasonable power allocation in a vehicular-to-vehicular (V2V) communication network, Zhang et al. [27] proposed a deep reinforcement learning method to choose the optimal relay according to the velocity, location, and packet number of each vehicle. In [16], He et al. proposed a Q-learning algorithm to find optimal UAV relays to assist V2V communication. Their proposed method improved the delivery ratio and delivery latency with comprehensive consideration of the state transition probability of communication interruption, delay consumption, and energy consumption.

However, these methods only consider the delay of a single hop and ignore the total delay of the whole link. In some cases, if we only consider the shortest delay of a single hop, it is likely to choose the closest next-hop relay. This causes the hop number of the whole link to increase, along with the total link latency. Meanwhile, most methods use an infinitely long cache during the computation of system throughput. However, in real life, the buffer size is limited. Therefore, the impact of packet loss on throughput must be considered.

## 3. System Model

**Figure 1:**
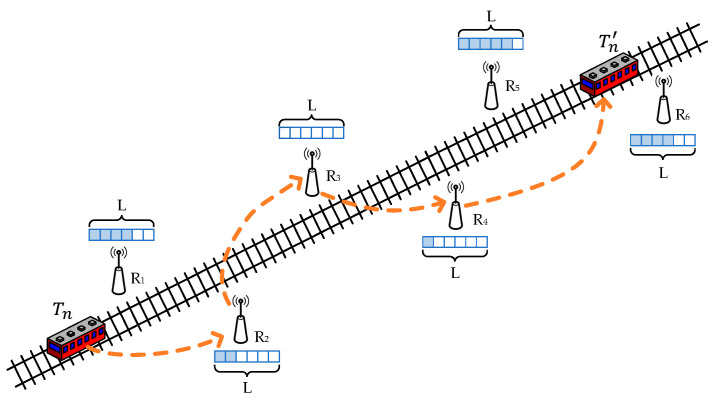
Next-hop relay selection in T2T communication.

As shown in Figure 1, we consider a T2T communication over a multi-hop wireless ad hoc network. In this scenario, since the coverage of one hop is very limited, the train needs the assistance of wayside relays for multi-hop transmission. There are multiple relays within the communication range of each train and wayside node; hence, they need to select the most suitable next-hop wayside relays among these candidate nodes. For example, $R_{2}$ may communicate with $R_{3}$, $R_{4}$, and $R_{5}$, but $R_{3}$ is chosen as the next hop node by considering factors such as channel quality and delay.

Therefore, for N trains running on the rail, denoted as $T=\left\{T_{1},T_{2},\ldots ,T_{n},\ldots T_{N}\right\},$ there are M wayside relays distributed beside the rail, denoted as $\;R=\left\{R_{1},R_{2},\ldots ,R_{m},\ldots R_{M}\right\}.$ The train has high mobility; in order to ensure the quality of the T2W transmission, we assume that there are two orthogonal frequency bands available: band 1 for train-to-wayside (T2W) transmission [28] and band 2 for wireless wayside-to-wayside (W2W) transmission. Since the transmission is on two orthogonal channels, there is no interference between T2W and W2W transmission, while multiple W2W transmissions at the same time will cause interference.

In multi-hop transmission, all relays follow the decode-and-forward (DF) principle. Furthermore, we assume that the whole system is stationary in the time slot t, and that the transmit power of the nodes does not change. All channels follow quasi-static Rayleigh fading, such that the channel gain between node a and node b can be represented as follows:

(1) $$h_{a,b}=X_{a,b}\;d_{a,b}^{-\beta /2},$$

where $h_{a,b}$ is the instantaneous channel gain of link a→b, $X_{a,b}$ is the fading coefficient, and $d_{a,b}$ and β indicate the distance between two nodes and the path-loss exponent [29].

### 3.1. Communication Model

#### 3.1.1. Train-to-Wayside (T2W) Link

The transmission of T2W link is on an independent channel; thus, there is no other link interference, and the signal-to-noise ratio (SNR) of the T2W transmission is

(2) $$\gamma_{T_{n},R_{m}}=\frac{p_{T_{n}}{|h_{T_{n},R_{m}}|}^{2}}{N_{0}},$$

where $p_{T_{n}}$ is the transmission power of the train $T_{n}$, $h_{T_{n},R_{m}}$ is the channel gain between the train $T_{n}$ and the wayside relay $R_{m}$, and $N_{0}$ is the noise power. Hence, the channel throughput [15] between train $T_{n}$ and the wayside relay $R_{m}$ is

(3) $$C_{T_{n},R_{m}}=B\log_{2}(1+\gamma_{T_{n},R_{m}}).$$

As for the final hop, wayside node $R_{m}$ transmits to destination train ${T^{\prime }}_{n}$, which is also calculated in the same way as above, except that the transmission direction is different. The throughput between $R_{m}$ and destination train ${T^{\prime }}_{n}$ can be expressed as

(4) $$C_{R_{m},{T^{\prime }}_{n}}=B\log_{2}(1+\gamma_{R_{m},{T^{\prime }}_{n}}).$$

#### 3.1.2. Wayside-to-Wayside (W2W) Link

At time slot t, it is possible that more than one wireless W2W link transmits information simultaneously; thus, there is interference between W2W links [16]. During the packet transmission, the one-hop link from wayside relay i to wayside relay j at time slot t is denoted as $l_{i,j}(t)$, where $\;i,j\in \left\{R_{1},R_{2},\ldots ,R_{m},\ldots R_{M}\right\}$. Moreover, $\rho_{i,j}(t)$ represents the i→j link transmission status. $\rho_{i,j}(t)=1$ denotes that wayside node i is transmitting with node j. Therefore, the SNR for the transmission of wayside node i and wayside node j can be represented as

(5) $$\gamma_{i,j}=\frac{P_{i}{\left|h_{i,j}\right|}^{2}}{N_{0}+\sum_{l_{i,j}(t)\neq l_{i^{\prime },j^{\prime }}(t)}\rho_{i^{\prime },j^{\prime }}P_{i^{\prime }}{\left|h_{i^{\prime },j^{\prime }}\right|}^{2}},$$

where $l_{i^{\prime },j^{\prime }}(t)$ is the interference link during the time slot t.

The transmission throughput between wayside relay i and wayside relay j is

(6) $$C_{i,j}=B\log_{2}(1+\gamma_{i,j}).$$

#### 3.1.3. Outage Analysis

In wireless networks, outage events occur when the actual mutual information is less than the required data rate [30]. To ensure the reliability of information transmission, the SNR of the channel must be greater than the SNR threshold value $\gamma_{th}$ to transmit. At time slot t, the transmission condition for train $T_{n}$ with wayside node $R_{m}$ is $\gamma_{T_{n},R_{m}}>\gamma_{th}$, and the transmission condition for wayside node i and wayside node j is $\gamma_{i,j}>\gamma_{th}$. In particular, the maximum transmission distance $R_{\max}$ between train and wayside relay while the train $T_{n}$ is moving can be calculated as

(7) $$\frac{p_{T_{n}}{\left|X_{T_{n},R_{m}}d_{T_{n},R_{m}}^{-\beta /2}\right|}^{2}}{N_{0}}\geq \gamma_{th}.$$

We can obtain the maximum distance $R_{\max}$ as

(8) $$R_{\max}=d_{T_{n},R_{m}}={\left(\frac{N_{0}}{p_{T_{n}}{X_{T_{n},R_{m}}}^{2}}\gamma_{th}\right)}^{-\frac{1}{\beta }}.$$

### 3.2. Optimal Relay Selection

#### 3.2.1. Mobile Reliable Model

Due to the mobility of trains, the train $T_{n}$ may move out of the communication range of the wayside relay $R_{m}$, resulting in an outage event; hence, the distance between the train and wayside nodes should be limited. The candidate wayside node locations are denoted as $\left(x_{R_{1}},y_{R_{1}}\right),\;\left(x_{R_{2}},y_{R_{2}}\right),\;\left(x_{R_{m}},y_{R_{m}}\right)\ldots \;\left(x_{R_{M}},y_{R_{M}}\right).$ In the transmission delay $T_{c}$ between train and wayside node, the channel SNR must satisfy the SNR threshold condition, whether the train is in the initial position $\left(x_{R_{1}},y_{R_{1}}\right)$ or at the end of transmission position $\left({x^{\prime }}_{T_{1}},{y^{\prime }}_{T_{1}}\right)$. Meanwhile, the speed of packet transmission in the channel is much faster than the speed of the train; thus, we assume that the train drives at initial speed v(t) during the transmission. In the transmission time delay $T_{c}$, the distance of the train moving in the x- and y-directions can be calculated as follows [15]:

(9) $$S_{x}=v(t)\times {\;T}_{c}\times e_{x}(t),$$

(10) $$S_{y}=v(t)\times T_{c}\times e_{y}(t),$$

where $e_{x}(t)$ and $e_{y}(t)$ are the x- and y-directions of the train. Therefore, the location where the train ends its transmission is $\left({x^{\prime }}_{T_{1}},{y^{\prime }}_{T_{1}}\right)=\left(x_{T_{1}}+{\;S}_{x},y_{T_{1}}+{\;S}_{y}\right).$ The conditions that candidate wayside nodes need to satisfy are

(11) $$d_{T1,Rm}=\sqrt{{\left(x_{T1}\;{-x}_{Rm}\right)}^{2}+{\left(y_{T1}\;{-y}_{Rm}\right)}^{2}}<R_{\max}\;,$$

(12) $${d^{\prime }}_{T1,Rm}=\sqrt{{\left({x^{\prime }}_{T1}\;{-x}_{Rm}\right)}^{2}+{\left({y^{\prime }}_{T1}\;{-y}_{Rm}\right)}^{2}}<R_{\max}\;.$$

A node can only be a candidate transmission node for a train if its location is within the transmission range of the train at the beginning and end of the transmission.

#### 3.2.2. Delay Model

During the packet transmission, a time delay is generated. In this section, we build a delay model to calculate the transmission delay between each node. We define the total delay $D_{i,j}$ of packet transmission between wayside nodes i,j into two main components, which are the transmission delay $T_{n}$ caused by the node sending the packet and the queuing delay $T_{q}$ due to node congestion. In this subsection, the time delay calculation is the same for both T2T transmission and T2W transmission; thus, both are expressed as the transmission between nodes a and b.

When the node sends data packets, the transmission delay can be represented as

(13) $$T_{c}=\frac{L}{C_{a,b}},$$

where L is the number of bits in the packet, and $C_{a,b}$ is the transmission rate of the channel between node a and node b.

Queuing delay [27] is unavoidable in the transmission of large amounts of data. Therefore, it is crucial to build a node queuing model. The queue follows the first-in first-out (FIFO) rule. When the CBTC system is stable, we assume that each wayside node can receive multiple data streams simultaneously to eliminate any scheduling effects, and that the average arriving rate and queuing situation are basically fixed. In order to calculate the queuing delay, we use Little’s formula.

In Little’s law, the average waiting time of a queue can be calculated as the queue length divided by the effective throughput. Since the buffer length of our designed model is limited, we calculate the effective throughput by considering the packet loss rate and the packet error rate. According to Little’s law [31,32], the packet delay at next-hop node b can be expressed as

(14) $$T_{q}=\frac{Q_{b}}{{Th}_{b}},$$

where $Q_{b}$ is the average number of packets queued at node b, and ${Th}_{b}$ is the effective throughput of node b.

The effective throughput of node b is indicated as

(15) $${Th}_{b}=\lambda_{b}\Delta t\times \;(1-p_{f}),$$

where $\lambda_{b}$ is the average arriving rate of node b, $\lambda_{b}\Delta t$ is the total number of packets arriving in a time slot Δt, and $p_{f}$ is the link a→b unsuccessful transmission rate. There are many factors that affect the transmission unsuccessful rate, such as the packet error rate $p_{fe}$ and the packet loss rate $p_{fl}$. If a packet is lost or transmission error occurs, this will cause this packet to be unusable; hence, the probability of unsuccessful transmission rate can be expressed as

(16) $$p_{f}=1-(1-p_{fl})(1-p_{fe}).$$

As for the calculation of the packet error rate, we can assume that the channel is modulated using the quadrature phase shift keying (QPSK) coding method. Therefore, the bit error rate (BER) [2] is

(17) $$p_{be}=\frac{1}{2}\left(1-\sqrt{\frac{\gamma_{a,b}}{1+\gamma_{a,b}}}\right),$$

where $\gamma_{a,b}$ is the SNR between the node a and node b; when the SNR between two nodes is large enough, $p_{be}≅\frac{1}{4\cdot \gamma_{a,b}}$. Meanwhile, as the length of the packet is L, the BER of the whole packet can be represented as

(18) $$p_{fe}=1-{\left(1-p_{be}\right)}^{L}.$$

For packet loss rate $p_{fl}$, we build a node queuing model to solve this problem [33,34]. Packet loss is due to the limited buffer length of the node. Therefore, if the total packet length exceeds the buffer length, the packet will not be received by the node, causing an increment in packet loss rate. We define M as the maximum number of packets that the buffer can hold, while $Q_{t-1}$ is denoted as the number of packets left in the previous time slot, and $A_{t}$ is the average number of packets arriving at the node in time slot t. We can derive the average number of arriving packets per time slot as $A_{t}=\lambda_{b}\Delta t$, where $\lambda_{b}$ is the arrival rate of node b, and L is the length of the packet. The level of packet loss is $F_{t}$, which can be expressed as

(19) $$F_{t}=\max[0,(Q_{t-1}+A_{t})-M].$$

When the train or wayside relay steadily sends packets to the next hop, $A_{t}$ and $F_{t}$ remain constant during transmission. Therefore, $\underset{t\to \infty }{\lim}A_{t}=A$ and $\underset{t\to \infty }{\lim}F_{t}=F$. In time slot t, the packet loss rate of this node can be calculated by

(20) $$p_{fl}=\underset{t\to \infty }{\lim}\frac{\sum_{t=1}^{T}F_{t}}{\sum_{t=1}^{T}A_{t}}=\frac{E\left\{F\right\}}{E\left\{A\right\}}=\frac{E\left\{F\right\}}{\lambda_{b}\Delta t},$$

where E{x} is the mathematical expectation. If there is no packet overflow, then the packet loss rate is zero. Otherwise, the packet loss rate is the number of packet losses divided by the number of node arrivals. The packet loss rate and the packet error rate are calculated in Equations (18) and (20), respectively, and then introduced into Equations (14) and (15) to calculate the queuing delay. Therefore, the total delay of the k hop from node a to node b can be expressed as

(21) $$D_{a,b}^{k}=T_{p}+T_{c}\;.$$

#### 3.2.3. Hop Tradeoff

In the next-hop selection, if we only pursue large throughput and small delay for one hop, this will result in an increase in the hop count of the entire T2T link. Therefore, we design a “hop tradeoff” indicator to optimize the number of hops on the entire link. The initial train $T_{n}$ and the destination train ${T^{\prime }}_{n}$ need to transmit information, and the distance of the whole T2T link is $d_{{T_{n},T^{\prime }}_{n}}$. During the transmission process, the distance between node a and node b for one-hop distance is $S_{a,b}$. We calculate the number of hops $k_{a,b}$ required to complete the entire T2T link for the one-hop distance $S_{a,b}$, which can be represented as

(22) $$k_{a,b}=\frac{d_{{T_{n},T^{\prime }}_{n}}}{S_{a,b}}\;.$$

## 4. Problem Formulation

In the CBTC scenario, there are different numbers of wayside nodes to assist information transmission depending on the distance between two trains; thus, the link selection for transmission is particularly important. To solve the problem of multi-hop relay selection in wireless ad hoc networks, we propose an optimal transmission model based on a discrete Markov process. The aim is to design a relay node selection decision that satisfies low latency and high throughput, so that information can be transmitted quickly and accurately between trains to each other.

Since the next-hop selection depends only on the current state and the next state changes with the current action selection, next hop selection can be considered as an MDP. The transfer probability between states in the MDP is unknown; thus, we can use the DRL approach to better solve our proposed problem. In DRL, the agent finds the optimal policy that maximizes the long-term reward value according to the channel state information (CSI) and the congestion level of the node. In this paper, we use multiagent DRL (MADRL), in which each node acts as an agent. As agents need to make decisions in a shorter period of time, the agent’s network is trained offline centrally by collecting information between nodes, and then porting it to each agent for online decision making. Therefore, each agent needs to select the next-hop node independently according to the current state, without additional communication for further training. In DRL, there are several key components, as described below.

(1) State Space

In each time slot, the agent updates and learns the policy by observing the variation of the state. In particular, the state contains two components: the number of packages queued in each node and the channel throughput of the links between the two nodes. In time slot t, the state space is defined as

(23) $$s_{t}=\left\{Q(t),\;C(t),\;V(t)\right\},$$

where Q(t) indicates the queue length of each node. C(t) is the throughput between the transmission node and other nodes. V(t)={0,1}; when V(t)=0, at time slot t, the wayside node sends packets, whereas V(t)=1 means that the train sends the packet.

(2) Action Space

According to the channel state and queue state of each candidate node, the optimal next-hop node A(t) is selected. The action space can be given by

(24) $$a_{t}=\left\{A(t)\right\},$$

where A(t)={0,1 … m … M}. If A(t)=m, then the next hop is node m.

(3) Reward Function

In the selection of the next-hop node, the optimization objective is to minimize the delay and maximum the throughput of the entire T2T link while ensuring that the next-hop SNR is greater than threshold value. The packet is successfully transmitted from the initial train to the destination train after k∈{1,2, … , K} hops through wayside nodes $\;i,j\in \left\{R_{1},R_{2},\ldots ,R_{m},\ldots R_{M}\right\}$. Furthermore, $T_{n}$ is the packet source train, and ${T^{\prime }}_{n}$ is the packet destination train.

- Overall Transmission Time of Whole Link

The total transmission time for packet transmission between source train $T_{n}$ and destination train ${T^{\prime }}_{n}$ is

(25) $$\tau_{{T_{n},T^{\prime }}_{n}=D_{T_{n},i}^{1}}+\sum_{k=2}^{K-1}D_{i,j}^{k}+D_{{j^{\prime },T^{\prime }}_{n}}^{K}\;.$$

- Throughput of Whole Link

In order to better measure the quality of each hop in the transmission link and considering the packet loss and packet error rate, the throughput of the entire link is defined as follows [35,36]:

(26) $$C_{{T_{n},T^{\prime }}_{n}}=(1-{p^{\prime }}_{f})\;\cdot \;\min\left\{E\left[C_{T_{n},i}\right],E\left[C_{i,j}\right]\ldots E\left[C_{{j^{\prime },T^{\prime }}_{n}}\right]\right\},$$

where ${p^{\prime }}_{f}$ is the unsuccessful transmission rate of whole link; $p_{f}$ for the one-hop unsuccessful rate is calculated using Equation (16). $C_{i,j}$ denotes the throughput between waysides node i and j.

- The Optimization Goal

The optimization goal for the proposed MADDQN is to reduce the latency and improve throughput for the whole link; hence, the proposed optimization goal is defined as

(27) $$\max\omega_{1}\frac{1}{\tau_{{T_{n},T^{\prime }}_{n}}}+\omega_{2}C_{{T_{n},T^{\prime }}_{n}}\;$$

$$\mathrm{s.}t:\;C1:\;\gamma_{i,j}^{k}>\gamma_{th}\;,$$

$$C2:\;D_{i,j}^{k}>D_{th}\;,$$

$$C3:\;d_{T_{n},i}<R_{\max}\;.$$

Here, $\tau_{{T_{n},T^{\prime }}_{n}}$ and $C_{{T_{n},T^{\prime }}_{n}}$ are the whole-link latency and throughput between train $T_{n}$ and train ${T^{\prime }}_{n}$, respectively. $\omega_{1}$ and $\omega_{2}$ are the weight factors of the delay and throughput ($\omega_{1}+\omega_{2}=1$). *C*1 is to ensure that the SNR of the channel is greater than threshold value. *C*2 indicates that each hop delay needs to be less than the target transmission time. If the one-hop delay takes too much time, the transmission is considered to fail. *C*3 indicates that, when the train is transmitted with the wayside node i, the distance should be less than the maximum transmission distance.

The defined reward function comprehensively considers the throughput and delay of each hop, as well as adds the indicator $k_{i,j}$ in Section 3.2.3. Therefore, the reward function is defined as

(28) $$F_{t}=\{\begin{array}{c} \omega_{1}\frac{1}{k_{i,j}D_{i,j}^{k}}+\omega_{2}C_{i,j}^{k}+r_{s}+r_{c},\;\;\;\;\;\;if\;C1–C3\;are\;satisfied\\ \omega_{1}\frac{1}{k_{i,j}D_{i,j}^{k}}+\omega_{2}C_{i,j}^{k}+r_{s},\;\;\;\;\;\;otherwise \end{array},$$

where $r_{s}$ is an additional reward for the final hop directly to the train. This reward is established to prevent other wayside nodes close to the train from being selected, which may lead to an increase in hops and delay. $r_{c}$ is the outage penalty caused by the next-hop node out of the communication range and a long single-hop delay under *C*1–*C*3. 

## 5. Problem Solution

Since value-based functions are suitable for solving discrete space problems, and next-hop relay selection is a discrete action, we choose a value-based reinforcement learning approach for policy optimization. Our proposed scheme has a large number of channel states and queuing states, which leads to a high dimension of the Q-table, and makes the Q-learning [37] algorithm difficult to coverage during training. However, the DQN algorithm can solve this problem, featuring a combination of a deep neural network (DNN) and Q-value. DQN does not directly select the action with the highest Q-value in the Q-table, but fits the $Q_{\pi }\left(s_{t},a_{t},\theta \right)$ through the neural network [38]. Compared to recording the Q-value for an all-action-state situation, DQN just needs to store the weights of each neuron to calculate the Q-values for all policies $\pi \left(s_{t},a_{t}\right)$, which greatly reduces the storage space and makes the algorithm converge faster [39,40].

**Figure 2:**
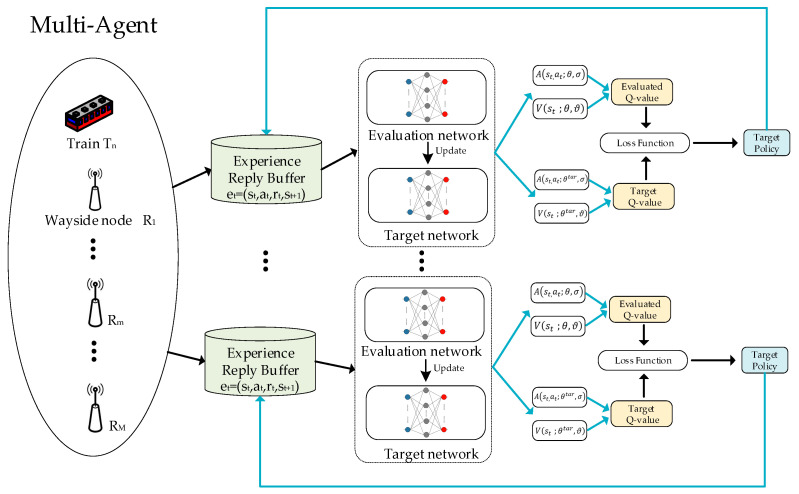
Framework of MADDQN.

Since each node needs to make the decision to select the next-hop node, we use the multiagent dueling DQN (MADDQN) approach. MADDQN treats each node as an independent agent, and all the agents are trained centrally. When making the next-hop selection, the trained network parameters are shared with all nodes, and each node selects the next-hop node individually. The specific process of MADDQN is shown in Figure 2.

### 5.1. DQN

In each time slot t, each node acts as an agent to observe the current state $s_{t}$ of the system, including the congestion level and channel state information of all nodes. Then, the agent chooses a suitable action $a_{t}$ to select the next-hop node. After selecting an action $a_{t}$, a new state $s_{t+1}$ is obtained, and the reward $r_{t}$ corresponding to this action is also computed. The goal of the agent is to find a policy $\pi \left(s_{t},a_{t}\right)$, which maximizes the expected discounted cumulative reward $E\left[\sum_{i=t}^{K-1}\gamma^{i-t}r_{i+1}\right]$ [9]. Therefore, the action-state value function is used to calculate the expected discounted cumulative reward of each relay selection policy and then select the policy π with the largest reward. The state-action value function is defined as

(29) $$Q_{\pi }\left(s_{t},a_{t}\right)≜E\left[\sum_{i=t}^{K-1}\gamma^{i-t}r_{i+1}|s_{t},a_{t},\pi \right],$$

where γ∈(0,1) is the discount factor, which represents the ratio between immediate and long-term reward. $r_{i+1}$ is the immediate reward at time slot i+1.

In the next time slot of action selection, not only is the next-hop relay selected with maximum $Q_{\pi }\left(s_{t},a_{t}\right)$, but the ε−greedy algorithm is also added to explore extra actions. In order to try more possible actions and avoid falling into the local maximum, the agent has ε possibility to choose an action randomly. The ε−greedy algorithm is denoted as

(30) $$a_{t}=\{\begin{array}{c} \arg\max Q_{\pi }\left(s_{t},a_{t}\right),\;\;\;\;with\;probability\;1-\varepsilon \\ random,\;\;\;\;\;\;\;\;\;\;\;\;\;\;\;\;\;\;\;with\;probability\;\varepsilon \end{array}.$$

Due to the different channel states and node congestion levels, a large number of states are formed; thus, it is impossible to calculate the Q-value for each action and state. In DQN, the convolutional neural network (CNN) is trained to get the weight θ of each neuron. After inputting the current channel state and the next-hop relay selection action, the neural network can fit a state-action value $Q\left(s_{t},a_{t},\theta \right)$. To make the network converge faster, DQN has two networks: the target network and the evaluation network. During the training process, the weights of the evaluation network θ are continuously updated, and the weights of the evaluation network are assigned to the target network $\theta^{tar}$ at certain time intervals. Then, the weight θ is updated by the stochastic gradient descent method to minimize the result of loss function between the target network and the evaluation network. The loss function between the target network and the evaluation network is defined as

(31) $$L\left(\theta \right)=E_{\left(s_{t},a_{t},r_{t},a_{t+1}\right)}{\left(Q_{t\arg et}-Q\left(s_{t},a_{t},\theta \right)\right)}^{2},$$

where the target value for each iteration of the network is represented as

(32) $$Q_{target}=r_{t}+\gamma \;\underset{a_{t+1}}{\max}Q\left(s_{t},a_{t},\theta^{tar}\right).$$

To focus more on historical experiences and disrupt the correlation between experiences, DQN also uses the mechanism of experience replay. At each time slot, when nodes are trained centrally, the node acts as an agent, storing the training experience $e_{t}=(s_{t},a_{t},r_{t},s_{t+1})$ into the experience pool, and then forming the sequence $D=\left\{e_{1},e_{2}\ldots e_{N}\right\}$. For each training, a small number of samples are randomly selected from the experience pool as a batch for network training, which makes the network converge better. 

### 5.2. Dueling DQN

The dueling DQN network [19] makes further improvements on the DQN network structure. In DQN, the network directly outputs the state-action values $Q\left(s_{t},a_{t},\theta \right)$ corresponding to each relay selection policy. However, in dueling DQN, the output Q-value is split into two branches: the state value $V\left(s_{t}\right)$ indicating the value of the current channel and queuing state, and the action advantage value $A\left(s_{t},a_{t}\right)$ representing the value brought by the relay selection action. Finally, the output values of the two branches are combined to make the estimation of Q more accurate. The combination of the two branches can be written as

(33) $$Q\left(s_{t},a_{t};\theta ,\sigma ,\vartheta \right)=V\left(s_{t};\theta ,\vartheta \right)+A\left(s_{t},a_{t};\theta ,\sigma \right),$$

where θ, σ, and ϑ are the coefficients of the neural network. In order to prevent multiple sets of state value $V\left(s_{t}\right)$ and action advantage value $A\left(s_{t},a_{t}\right)$ with the same state-action value $Q\left(s_{t},a_{t};\theta ,\sigma ,\vartheta \right)$, and to make the algorithm more stable [38], Equation (30) is replaced by

(34) $$Q\left(s_{t},a_{t};\theta ,\sigma ,\vartheta \right)=V\left(s_{t};\theta ,\vartheta \right)+\left(A\left(s_{t},a_{t};\theta ,\sigma \right)-\frac{1}{\left|A\right|}\sum_{a_{t+1}}A\left(s_{t},a_{t+1};\theta ,\sigma \right)\right).$$

The proposed multiagent dueling DQN is shown in Algorithm 1.
**Algorithm 1** Dueling-DQN1: Initialization: Initialize the maximum buffer capacity M and packet length L; Initialize the number of nodes along the rail N; Initialize network memory size J, batch size B, greedy coefficient ε, and learning rate φ. 2: **for** episode in range K do: 3:    Reset channel quality C and the queue length Q of each node as initial state $S_{initial}$ 4:    **While**
a(t)!=Destination Node
**do** 5:      Choose action: with probability ε to choose next hop node in random. 6:                    Otherwise, choose action $a_{t}$ with ${\arg\max Q}_{\pi }\left(s_{t},a_{t},\theta \right)$. 7:       From current state $s_{t}$ and action $a_{t}$ of this hop, obtain the reward $r_{t}$ for this action $a_{t}$ and the next state $s_{t+1}$. 8:       Store $\langle s_{t},a_{t},r_{t},s_{t+1}\rangle$ into experience reply to memory. 9:       Randomly take minibatch of $\langle s_{t},a_{t},r_{t},s_{t+1}\rangle$ from experience reply to memory. 10:     Combine two branches $V\left(s_{t};\theta ,\vartheta \right)$ and $A\left(s_{t},a_{t};\theta ,\sigma \right)$ into $Q\left(s_{t},a_{t};\theta ,\sigma ,\vartheta \right)$ 11:     Calculate target Q-value $Q_{target}=\{{}_{r_{t}+\gamma \;\underset{a_{t+1}}{\max}\;Q(s_{t+1},a_{t+1};\theta^{tar},\sigma ,\vartheta ),\;\;\;\;\;\;\;otherwise.}^{r_{t},\;\;\;\;\;\;\;\;\;\;\;\;\;\;\;\;\;\;\;\;\;\;\;\;\;\;\;\;\;\;\;\;\;\;\;\;\;\;\;\;\;\;\;\;\;\;\;\;\;\;\;\;\;\;\;if\;a_{t}\;is\;the\;destination\;node}$ 12:     Minimize loss function L(θ) using Equation (30) 13:   Update the target network after several steps using the parameters of the evaluation network14:  **end while** 15: **end for**

## 6. Simulation Results

In this section, we verify the effectiveness of the proposed deep learning-based relay selection algorithm by conducting simulation experiments in CBTC system.

### 6.1. Simulation Settings

In the simulation, TensorFlow 1.13.1 was imported in Python 3.6 as the simulation environment.

In order to simplify the system model, we performed a simulation of relay selection between two adjacent trains. If the SNR between the current node and the next-hop wayside relay is greater than the SNR threshold, then the communication between the two nodes is possible. Since each node has the same transmission power, the distance between nodes mainly determines the channel throughput; thus, the next-hop node which is closer to the current node has higher one-hop channel throughput. In the train system, packets transmitted by the train must pass through wayside nodes and cannot be delivered directly to the forward train. In addition, wayside relays are uniformly distributed on both sides of the track, as the distance between trains become longer, the number of hops required for transmission also increases.

Furthermore, in the process of training, each agent has its own training parameters; we set the batch size B=256, greedy coefficient ε=0.1, learning rate φ=0.001, and memory size J=1024. Some other main parameters of the communication system are shown in Table 1.

### 6.2. Performance Analysis

We compare the proposed MADDQN algorithm with two existing algorithms:

1. GPSR [22] (greedy perimeter stateless routing) is often used in the transmission of ad hoc networks, which collects the geographic location information of neighboring nodes and finds the next-hop node with the nearest geographic location to the destination through a greedy algorithm.
2. The random selection scheme randomly selects the next-hop node within the communication range without any optimization strategy.

#### 6.2.1. Performance Comparison of Convergence

**Figure 3:**
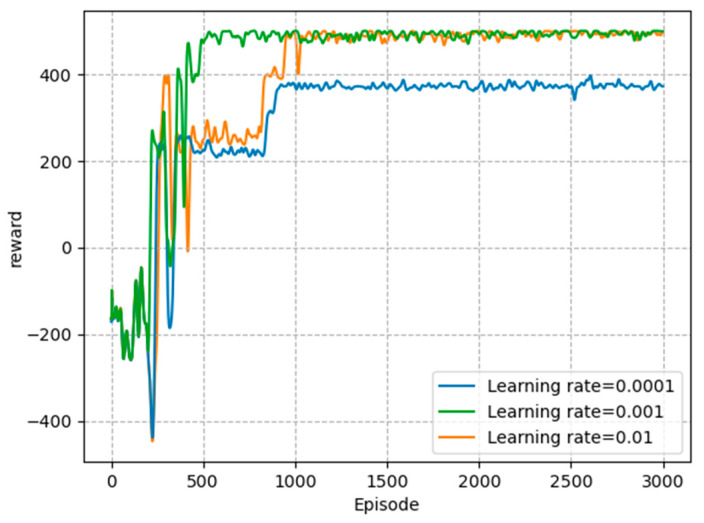
Total reward with different learning rates.

Firstly, in order to find the learning rate that makes the proposed model converge best, we conducted experiments at three different learning rates. As shown in Figure 3, when the learning rate was equal to $10^{-4}$, the convergence rate of the agent was slow, and the total reward value did not reach the optimal value. To make the convergence speed up, we increased the learning rate to $10^{-3}$, which made the convergence faster and the total reward higher. When the learning rate increased to $10^{-2}$, it was easy for the agent to converge to a local optimum, resulting in poor convergence results. Therefore, in the training of the agents, the learning rate was set to $10^{-3}$. 

**Figure 4:**
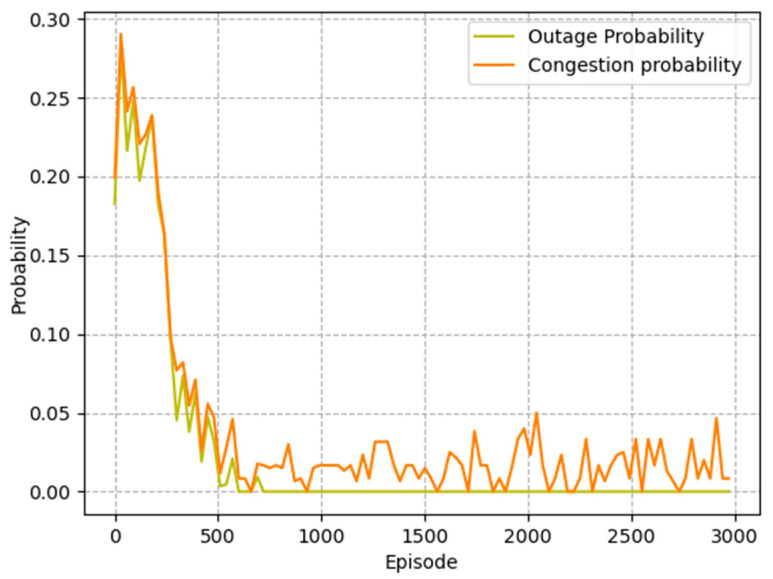
Outage probability and congestion probability of the proposed algorithm.

The goal of agent training is to better avoid outage events and reduce packet loss rate. As shown in Figure 4, the probability of outage events decreased dramatically in the first 1000 episodes, indicating that the agent learned to select next-hop relays within communication range. At the same time, the probability of network congestion gradually decreased during the training process, which illustrates that the agent successfully avoided congested nodes. The simulation results show that the MADDQN algorithm could effectively avoid outage and congestion events, ensuring the quality of transmission.

#### 6.2.2. Performance Comparison of Different Aspects

**Figure 5:**
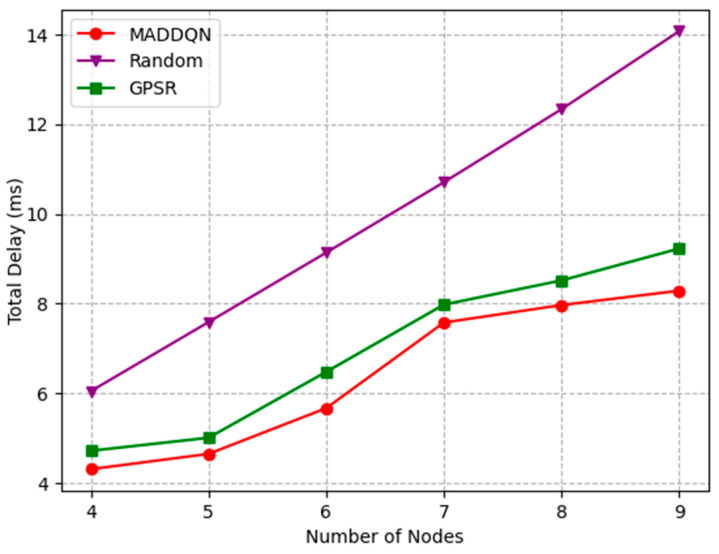
Total delay versus the number of nodes.

The distance between two adjacent trains is different and the wayside nodes are uniformly distributed. Thus, when the distance between two trains become larger, the number of trackside nodes between them increases and the topology of the network changes. Figure 5 depicts the curves of the variation of the total delay as the number of nodes increase. It can be observed that the whole-link delay increased from 4.31 ms to 8.43 ms under the MADDQN algorithm. The main reason is that, with the increment in the number of relays, the number of hops required for the whole link increased; thus, the total delay increased. Compared with the random selection scheme and GPSR algorithm, the transmission delay of the MADDQN algorithm was reduced by an average of 2 ms and 0.5 ms, respectively. Although the traditional GPSR algorithm requires a small number of hops, it cannot avoid congested nodes, resulting in a large packet loss rate. In addition, the random scheme can neither select nodes with small queued tasks nor optimize the hop count; hence, the delay is longer than the previous two algorithms.

**Figure 6:**
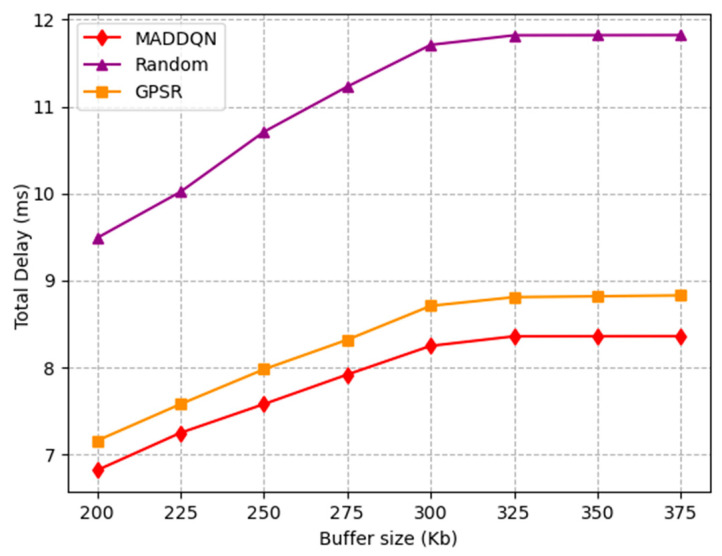
Total delay versus buffer size.

The effect of buffer size on total delay is investigated in Figure 6. When the buffer size was less than 300 Kb, the total latency increased significantly as the buffer size became larger. When the buffer size was small, the packet queue became shorter, resulting in lower packet delay. Moreover, when the buffer size reached 300 kb, the total delay gradually flattened out. The reason is that nodes were no longer dropping packets, and the queue length of each node tended to be stable. In addition, MADDQN improved by 0.5 ms compared to the GPSR algorithm and 3 ms compared to the random selection scheme, which illustrates that the proposed MADDQN algorithm could select nodes with shorter queues for transmission.

**Figure 7:**
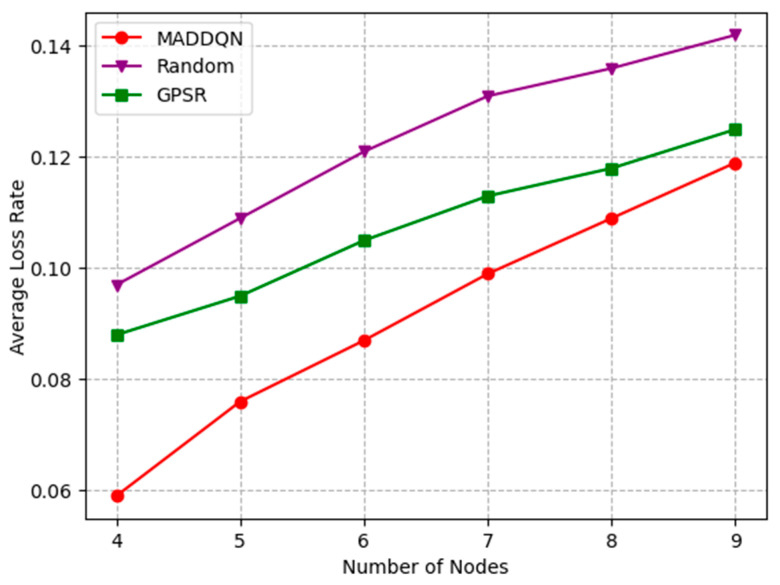
Average loss rate versus the number of nodes.

Figure 7 presents the relationship between the number of nodes and the average loss rate under different schemes. As the number of nodes increased from four to nine, the packet loss rate of MADDQN increased to 0.12. The reason is that, as the number of hops increased, the total packet loss rate also increased. Compared with the other two schemes, the proposed MADDQN could select the next-hop relay with a shorter queuing number for transmission, which greatly reduced the packet loss probability. While the GPSR algorithm could not avoid congested nodes, it required fewer hops for transmission; hence, the packet loss rate was also lower than the random scheme.

**Figure 8:**
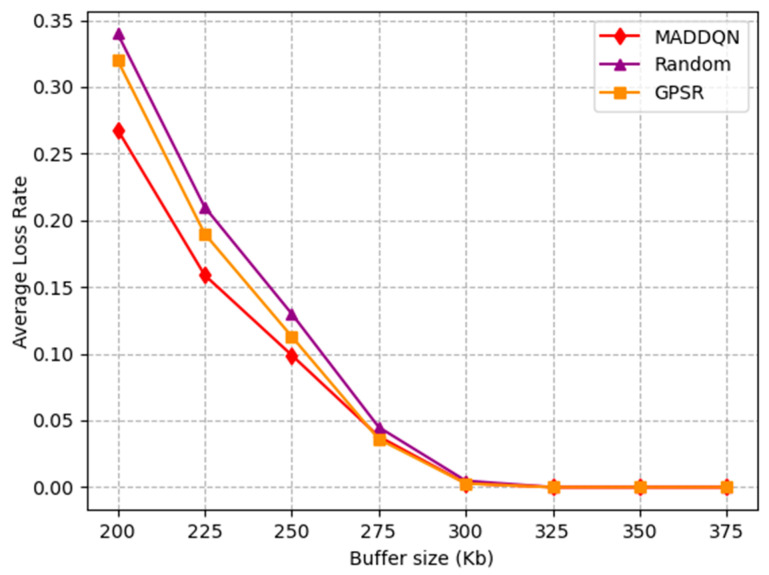
Average loss rate versus buffer size.

Figure 8 depicts the change in the average loss rate with the buffer size. The average packet loss rate rapidly decreased to zero when the buffer size was 300 kb. Due to the small buffer size, packets could easily overflow. Hence, the packet loss rate continued to decrease until the buffer was large enough. The average loss rate of the GPSR and random scheme before 300 kb was higher than MADDQN, which illustrates that our proposed method had a significant effect in avoiding congested nodes and reducing the number of hops, such that the packet loss rate was the lowest.

**Figure 9:**
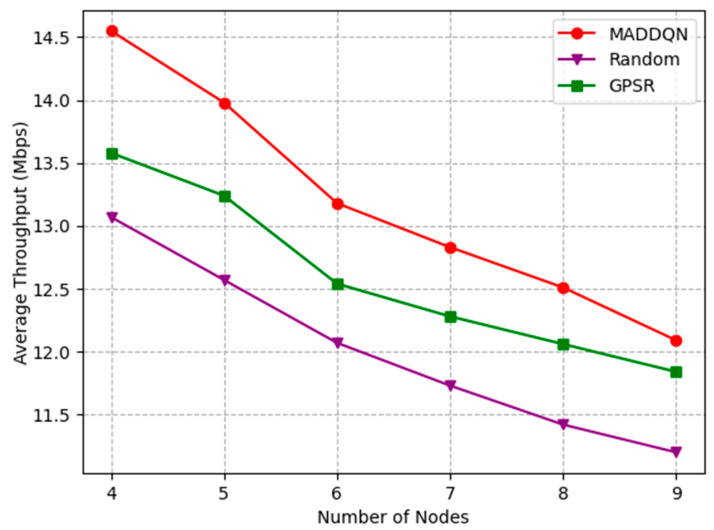
Average throughput versus the number of nodes.

Figure 9 presents how the number of nodes affects the average throughput. It can be observed that the average throughput of the entire link decreased monotonically with the increment in the number of nodes. This is because, as shown in Figure 7, the packet loss rate increased with the number of nodes, it led to a reduction in the overall throughput. The throughput of MADDQN was greater than the other two methods, indicating that, when selecting the next hop node, MADDQN chose the node with relatively large channel throughput and fewer queued packets, ensuring channel quality.

**Figure 10:**
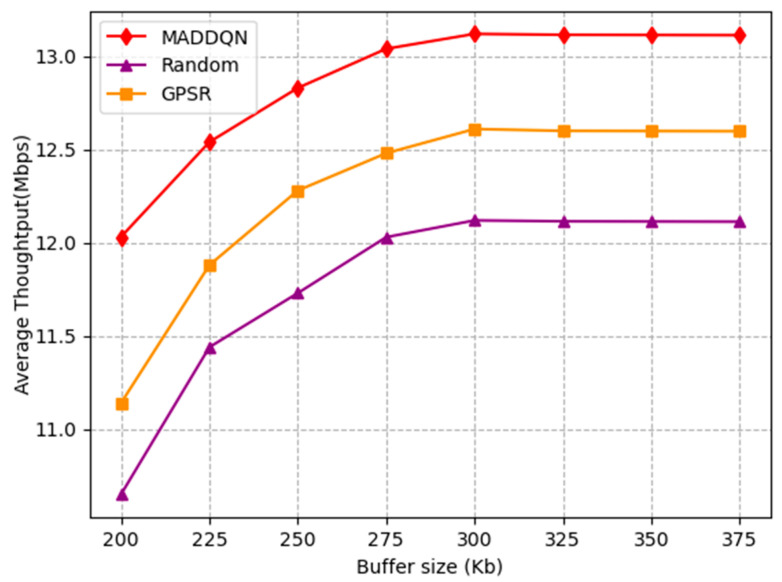
Average throughput versus buffer size.

Figure 10 shows the impact of buffer size on the throughput. It can be observed that the throughput rapidly increased before 300 kb and then reached a stable state with increasing buffer size. This is because as the buffer size gradually increased, it caused a decrease in packet loss rate; therefore, the system throughput increased. When the system had no packet loss, the throughput tended to stable. Meanwhile, the proposed algorithm had the highest throughput compared with the other two schemes; hence, it can be proven that MADDQN was effective in selecting the routing with a large throughput.

**Figure 11:**
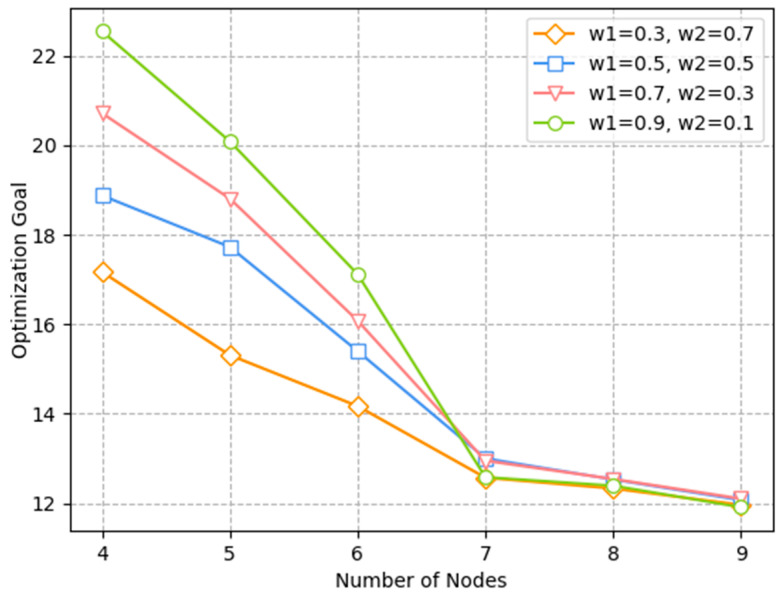
Relationship of optimization goal and number of nodes under different weights.

Figure 11 illustrates the relationship of the number of nodes and optimization goal under different weights of latency and channel throughput. The simulation results show that, with the rise in $\omega_{1}$, the optimization goal was much larger. This is because, although the optimization goal optimized both delay and throughput, when the weight of delay was high, latency was optimized more, while the optimization of throughput was relatively weak. Moreover, when the node number was between four and six, delay accounted for a large proportion of the optimization objective. Thus, when $\omega_{1}$ = 0.9, the total optimization objective was the highest. However, in order to optimize both delay and throughput to a better level, we chose the case $\omega_{1}$ = 0.5, $\omega_{2}$ = 0.5. In the application, different parameters can also be chosen according to different needs. For example, the weight of $\omega_{1}$ can be increased for safety information with higher requirements on latency. For systems where throughput is more important, the proportion of $\omega_{2}$ can be increased appropriately, but the sum of $\omega_{1}$ and $\omega_{2}$ must be equal to 1.

**Figure 12:**
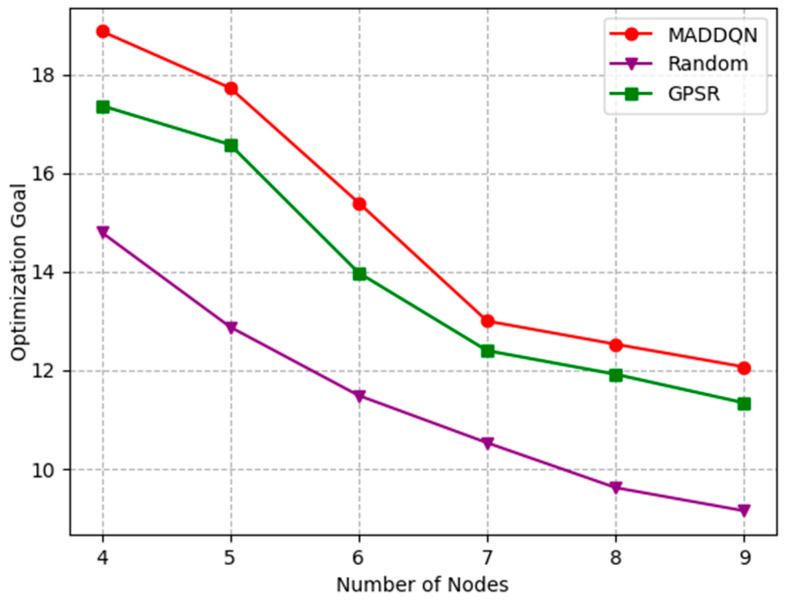
Relationship of optimization goal and number of nodes under different schemes.

**Figure 13:**
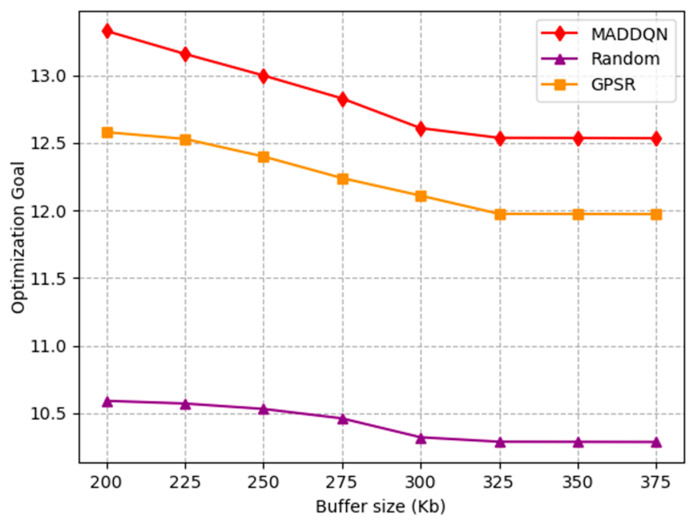
Relationship of optimization goal and buffer size under different schemes.

The effect of the number of nodes and the buffer size on the optimization goal is investigated in Figure 12 and Figure 13. The optimization goal was derived from Equation (27), which is a comprehensive indicator of channel throughput and transmission delay. In Figure 12, the delay increased and the throughput decreased as the number of nodes grew; thus, the optimization objective was gradually reduced. This shows that, as the number of nodes increased, the overall performance of the system worsened. In Figure 13, both latency and throughput tended to rise as the buffer size increased, but latency rose faster, having a greater impact on the optimization objective. Therefore, the optimization goal showed a slight decrease after combining these two indicators. Moreover, we can observe that our proposed algorithm always outperformed the existing algorithms, indicating that the MADDQN algorithm could better tradeoff the channel throughput and transmission delay under any topological condition and buffer size to achieve the optimization goal.

## 7. Conclusions and Future Work

In this paper, we designed a multi-hop relay selection strategy based on wireless ad hoc networks to assist T2T communication. The optimization goal of our proposed algorithm is to reduce the T2T transmission delay and increase the throughput of the entire link in a congested network. Since the channel status changes in real time, an MADDQN approach was proposed to better solve the problem. Simulation results showed that our proposed algorithm could effectively avoid congested nodes and reduce the number of hops for the whole-link transmission, thereby better achieving the optimization goal compared with existing routing algorithms. In future work, the energy consumption of the nodes and the problem of retransmission after packet loss should be considered. Moreover, some secure and energy-efficient technologies, such as reconfigurable intelligent surfaces (RIS), will be applied to the CBTC system to better assist in signal transmission.

## Figures and Tables

**Table 1:** Communication system parameters.

Parameters	Value
Number of trains	2
Number of wayside nodes	7
Bandwidth	10 MHz
Max buffer size	250 kb
Average packet size	25 kb
SNR threshold	31
Gaussian noise power spectral density	−174 dBm/Hz
Weight of latency, $\omega_{1}$	0.5
Weight of channel throughput, $\omega_{2}$	0.5

## Data Availability

Not applicable.

## References

[1] Wang X., Liu L., Tang T., Sun W. (2019). Enhancing Communication-Based Train Control Systems Through Train-to-Train Communications. IEEE Trans. Intell. Transp. Syst..

[2] Zhu L., Yu F.R., Ning B., Tang T. (2014). Communication-Based Train Control (CBTC) Systems with Cooperative Relaying: Design and Performance Analysis. IEEE Trans. Veh. Technol..

[3] Liu L., Parag P., Tang J., Chen W.Y., Chamberland J.F. (2007). Resource Allocation and Quality of Service Evaluation for Wireless Communication Systems Using Fluid Models. IEEE Trans. Inf. Theory..

[4] Wang X., Liu L., Tang T. (2020). Train-Centric CBTC Meets Age of Information in Train-to-Train Communications. IEEE Trans. Intell. Transp. Syst..

[5] Sun W., Yu F.R., Tang T., Bu B. (2016). Energy-Efficient Communication-Based Train Control Systems with Packet Delay and Loss. IEEE Trans. Intell. Transp. Syst..

[6] Wang X., Liu L., Tang T., Zhu L. Next Generation Train-Centric Communication-Based Train Control System with Train-to-Train (T2T) Communications. Proceedings of the 2018 International Conference on Intelligent Rail Transportation (ICIRT).

[7] Li Y., Zhu L. Collaborative Cloud and Edge Computing in 5G based Train Control Systems. Proceedings of the 2022 IEEE Global Communications Conference.

[8] Gong S., Lu X., Hoang D.T., Niyato D., Shu D., Shu L., Kim D.I., Liang Y.C. (2020). Toward Smart Wireless Communications via Intelligent Reflecting Surfaces: A Contemporary Survey. IEEE Commun. Surv. Tutor..

[9] Ahmed M., Wahid A., Laique S.S., Khan W.U., Ihsan A., Xu F., Chatzinotas S., Han Z. (2023). A Survey on STAR-RIS: Use Cases, Recent Advances, and Future Research Challenges. IEEE Internet Things J..

[10] Ahmed M., Mirza M.A., Raza S., Ahmad H., Xu F., Khan W.U., Lin Q., Han Z. (2023). Vehicular Communication Network Enabled CAV Data Offloading: A Review. IEEE Trans. Intell. Transp. Syst..

[11] Gupta L., Jain R., Vaszkun G. (2016). Survey of Important Issues in UAV Communication Networks. IEEE Commun. Surv. Tutor..

[12] Jacquet P., Muhlethaler P., Clausen T., Laouiti A., Qayyum A., Viennot L. Optimized Link State Routing Protocol for Ad Hoc Networks. Proceedings of the 2001 IEEE International Multi Topic Conference.

[13] Bai F., Sadagopan N., Helmy A. IMPORTANT: A framework to systematically analyze the Impact of Mobility on Performance of Routing Protocols for Adhoc Networks. Proceedings of the 2003 Twenty-Second Annual Joint Conference of the IEEE Computer and Communications Societies.

[14] Ding R., Xu Y., Gao F., Shen X. (2022). Trajectory Design and Access Control for Air–Ground Coordinated Communications System with Multiagent Deep Reinforcement Learning. IEEE Internet Things J..

[15] Ding R., Chen J., Wu W., Liu J., Gao F., Shen X. (2022). Packet Routing in Dynamic Multi-Hop UAV Relay Network: A Multi-Agent Learning Approach. IEEE Trans. Veh. Technol..

[16] He Y., Zhai D., Jiang Y., Zhang R. (2020). Relay Selection for UAV-Assisted Urban Vehicular Ad Hoc Networks. IEEE Wirel. Commun. Lett..

[17] Wu Q., Zheng J. Performance Modeling and Analysis of the ADHOC MAC Protocol for VANETs. Proceedings of the IEEE International Conference on Communication (ICC’15).

[18] Wu Q., Zheng J. (2016). Performance Modeling and Analysis of the ADHOC MAC Protocol for Vehicular Networks. Wirel. Netw..

[19] Wu Q., Zhao Y., Fan Q., Fan P., Wang J., Zhang C. (2023). Mobility-Aware Cooperative Caching in Vehicular Edge Computing Based on Asynchronous Federated and Deep Reinforcement Learning. IEEE J. Sel. Top. Signal Process..

[20] Zhu L., Yu F.R., Ning B. Availability Improvement for WLAN-Based Train-Ground Communication Systems in Communication-Based Train Control (CBTC). Proceedings of the 2010 IEEE 72nd Vehicular Technology Conference.

[21] Wang Y., Zhu L., Zhao H. Handover Performance Test and Analysis in TD-LTE based CBTC Train Ground Communication Systems. Proceedings of the 2017 Chinese Automation Congress.

[22] Karp B., Kung H.-T. Gpsr: Greedy Perimeter Stateless Routing for Wireless Networks. Proceedings of the 6th Annual International Conference on Mobile Computing and Networking.

[23] Liu K., Niu K. A Hybrid Relay Node Selection Strategy for VANET Routing. Proceedings of the 2017 IEEE/CIC International Conference on Communications in China (ICCC).

[24] Li F., Wang Y. (2007). Routing in Vehicular Ad Hoc Networks: A Survey. IEEE Veh. Technol. Mag..

[25] Toor Y., Muhlethaler P., Laouiti A., La Fortelle A.D. (2008). Vehicle Ad Hoc Networks: Applications and Related Technical Issues. IEEE Commun. Surv. Tutor..

[26] Wang Z., Han R., Li H., Knoblock E.J., Apaza R.D., Gasper M.R. Deep Reinforcement Learning Based Routing in an Air-to-Air Ad-hoc Network. Proceedings of the 2022 IEEE/AIAA 41st Digital Avionics Systems Conference (DASC).

[27] Zhang H., Chong S., Zhang X., Lin N. (2020). A Deep Reinforcement Learning Based D2D Relay Selection and Power Level Allocation in mmWave Vehicular Networks. IEEE Wirel. Commun. Lett..

[28] Wu Q., Zheng J. Performance Modeling of IEEE 802.11 DCF Based Fair Channel Access for Vehicular-to-Roadside Communication in a Non-Saturated State. Proceedings of the IEEE International Conference on Communication (ICC’14).

[29] Chen Y., Feng Z., Xu D., Liu Y. Optimal Power Allocation and Relay Selection in Dual-Hop and Multi-Hop Cognitive Networks. Proceedings of the 2012 IEEE International Conference on Communications (ICC).

[30] Wang Y., Feng Z., Chen X., Li R., Zhang P. Outage Constrained Power Allocation and Relay Selection for Multi-Hop Cognitive Network. Proceedings of the 2012 IEEE Vehicular Technology Conference (VTC Fall).

[31] Kleinrock L. (1975). Queueing Systems, Volume I: Theory.

[32] Liu W., Zhou S., Giannakis G.B. (2005). Queuing with Adaptive Modulation and Coding over Wireless Links: Cross-Layer Analysis and Design. IEEE Trans. Wirel. Commun..

[33] Ma R., Chang Y.-J., Chen H.-H., Chiu C.-Y. (2017). On Relay Selection Schemes for Relay-Assisted D2D Communications in LTE-A Systems. IEEE Trans. Veh. Technol..

[34] Chen Z., Smith D. (2021). MmWave M2M Networks: Improving Delay Performance of Relaying. IEEE Trans. Wirel. Commun..

[35] Xia B., Fan Y., Thompson J., Poor H.V. (2008). Buffering in a Three-Node Relay Network. IEEE Trans. Wirel. Commun..

[36] Gui J., Deng J. (2018). Multi-Hop Relay-Aided Underlay D2D Communications for Improving Cellular Coverage Quality. IEEE Access.

[37] Liu M., Yu F.R., Teng Y., Leung V.C.M., Song M. (2019). Performance Optimization for Blockchain-Enabled Industrial Internet of Things (IIoT) Systems: A Deep Reinforcement Learning Approach. IEEE Trans. Ind. Inf..

[38] Wang Z., Freitas N.D., Lanctot M. (2016). Dueling Network Architectures for Deep Reinforcement Learning. PMLR.

[39] Du J., Cheng W., Lu G., Cao H., Chu X., Zhang Z., Wang J. (2022). Resource Pricing and Allocation in MEC Enabled Blockchain Systems: An A3C Deep Reinforcement Learning Approach. IEEE Trans. Netw. Sci. Eng..

[40] Du J., Yu F.R., Lu G., Wang J., Jiang J., Chu X. (2020). MEC-Assisted Immersive VR Video Streaming over Terahertz Wireless Networks: A Deep Reinforcement Learning Approach. IEEE Internet Things J..

